# How Approaching Angle, Bottleneck Width and Walking Speed Affect the Use of a Bottleneck by Individuals

**DOI:** 10.3390/s24061720

**Published:** 2024-03-07

**Authors:** Ann Katrin Boomers, Maik Boltes, Uwe G. Kersting

**Affiliations:** 1 Institute for Advanced Simulation 7, Civil Safety Research, Forschungszentrum Jülich, 52428 Jülich, Germany; 2 Neuromechanics and Musculoskeletal Biomechanics, Institute of Biomechanics and Orthopaedics, German Sport University Cologne, 50933 Cologne, Germany

**Keywords:** bottleneck, pedestrian movement, 3D motion capturing, shoulder rotation, bottleneck crossing

## Abstract

Understanding pedestrian dynamics at bottlenecks and how pedestrians interact with their environment—particularly how they use and move in the space available to them—is of safety importance, since bottlenecks are a key point for pedestrian flow. We performed a series of experiments in which participants walked through a bottleneck individually for varying combinations of approaching angle, bottleneck width and walking speed, to investigate the dependence of the movement on safety-relevant influencing factors. Trajectories as well as 3D motion data were recorded for every participant. This paper shows that (1) the maximum amplitude of shoulder rotation is mainly determined by the ratio of the bottleneck width to the shoulder width of the participant, while the direction is determined by the starting angle and the foot position; (2) the ‘critical point’ is not invariant to the starting angle and walking speed; (3) differences between the maximum and minimum speed values arise mainly from the distribution of deceleration patterns; and (4) the position of crossing shifts by 1.75 cm/10 cm, increasing the bottleneck width in the direction of origin.

## 1. Introduction

Understanding pedestrian dynamics is of crucial relevance to the design of pedestrian facilities. This is applied, for example, when writing building regulations and manuals, managing crowds in event settings and developing or improving models that help to design well-functioning and safe facilities. Although pedestrian flows are part of everyday life, many fundamental properties of pedestrian dynamics cannot yet be described in full detail. Well-defined and thus reproducible laboratory experiments allow the targeted investigation of individual parameters influencing the dynamics and aid the development of model ideas, as well as the provision of precise data for the calibration and validation of models. While variables such as density and flow are used as a reference for the overall performance of a pedestrian model, it is only individual walking paths (trajectories), for example, that allow a microscopic view of the density distribution, which is important for safety in a crowd [1,2].

Even when considering trajectories, not all phenomena in pedestrian dynamics can be covered and no sufficiently precise models be developed that can describe, for example, clogging and its dissolution at bottlenecks [3,4], due to the fact that models are usually based on two dimensions and thus are not suitable for the description of three-dimensional processes without approximations [5,6,7]. Knowing the dynamics of people crossing a bottleneck is of safety importance, as bottlenecks are a key point for pedestrian flows. Various influencing factors are regularly identified, such as the freedom of movement or density, the motivation of the people, the external structural boundary conditions and the characteristics of the group of people. In order to map space requirements realistically, the relationships between space utilization and available space must be understood.

Space utilization is described by the movement of the body in space. To date, the literature on pedestrian dynamics has focused primarily on aspects of dynamics in confined areas, neglecting individual full body motion. A few studies deal with individual aspects of body motion, such as upper body rotation to describe avoidance behavior, but few in confined areas [6,8,9]. Individual movement as a function of structural obstacles, and individual positions with regard to obstacles or speed, on the other hand, have been addressed in biomechanics and sports medicine. Such studies are mostly limited to selected motion sequences of individual actions. For instance, the gait pattern of a human being walking in a straight line is described in detail in [10], including the phasing of body segments such as the upper body rotation (thorax) in the step cycle. The natural gait pattern shows a counter-clockwise rotation during a left foot swing and a clockwise rotation during a right foot swing. In [11], the speed dependence of upper body rotation is tested between 2 and 7 km/h at six speeds and it is found that there is little change in the amplitude or temporal occurrence of the maxima in the step cycle. Again, this study is performed only for straight walking and also shows that the phase between shoulder and hip rotation changes by up to 37% from in-phase to counter-phase. Other studies that have investigated these changes in the locomotor system for different speeds are [12,13,14,15,16]. Walking around a corner has been studied for three speeds for a 90° angle by [17]. It has been described that pedestrians walk closer to the obstacle around the corner the faster they walk. Several studies have considered the passage through a bottleneck. However, mostly either the speed was varied [18,19,20] or neither the speed nor the angle was varied due to other primary questions [21,22,23,24,25]. The parameters described are, e.g., a top-down strategy to be observed [11,14,18,26,27,28,29], such as between head and shoulder rotation, head rotation and the change in movement direction with delay times of 0.1 s to 0.6 s depending on the publication and locomotor parts included, as well as the beginning of the rotation of the head or shoulders at a constant distance (0.6 m to 1.1 m) in front of the obstacle [14,25].

An often used parameter is the amplitude of the shoulder rotation as a function of the bottleneck (aperture) width or the ratio of the bottleneck width *w* to the individual shoulder width *s* of a person (R=w/s) [18,19,22], which shows a larger amplitude the smaller the ratio. Warren and Whang [18] coined the term ‘critical point’, obtaining $R_{crit}$ = 1.3, which states that a person will rotate their shoulders to pass a bottleneck if the ratio is smaller than this value, and rotation is not necessary if the ratio is larger. Furthermore, a speed dependence (a larger amplitude the faster the person is walking [19]) or the absence of it [18], an age dependence and a dependence on the nature of the obstacle [22,23] have been described. Lastly, many studies refer to the value of 1.3 when setting obstacle widths when rotation is to be ensured [20,21,24,25,30].

The goal of this paper is to make knowledge from biomechanics applicable to pedestrian dynamics by describing the space requirements of individuals crossing a bottleneck using a dataset of an experimental series in which the bottleneck width, starting (approaching) angle and speed were varied, and to investigate the relationships between space and motion. The presented dataset closes the gap left by earlier studies, which varied individual parameters but never the three parameters described here simultaneously. Furthermore, this paper will also serve as a basis for future works to investigate how the described parameters change when several people encounter each other in a bottleneck, and how the utilization of space is adapted due to the interaction as well as the change in available space.

## 2. Experimental Setup and Procedures

To investigate the research aims raised in the previous section, we conducted bottleneck experiments with unhindered individuals. The experiments were carried out in June 2020 at the Research Center Jülich. A convenience sample of 13 coworkers was selected as participants in this study (Table 1). The inclusion criterion was a body size between 1.5 m and 2.0 m, to ensure that the person was fully seen by the camera and not obscured by the bottleneck construction, as well as a self-assessed moderate fitness level that would allow participants to be on their feet all day long.

**Figure 1:**
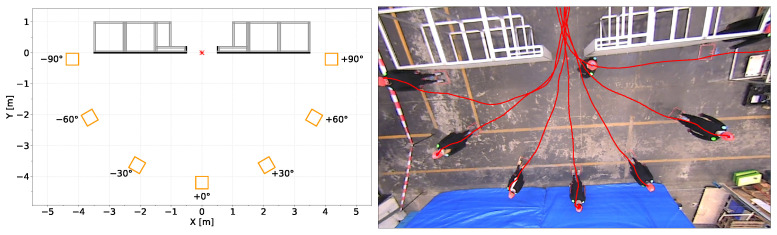
(**left**) Sketch of experimental setup and coordinate system exemplary for bottleneck width of 1.0 m. Bottleneck geometry is given in grey. Orange squares denote starting areas of participants. Red star denotes center of bottleneck. (**right**) Snapshot of participant walking individually from starting position at angle of +90° in software PeTrack [32]. Trajectories for each person visible in the snapshot are displayed as overlay in red.

The bottleneck construction consisted of two 3 m × 2 m × 1 m aluminium frames with gray plastic panels (Figure 1(right)), weighing 200 kg per side. The length of the bottleneck was adjustable in three steps (0.2 m, 1.0 m and 2.0 m). Since no dependence on length could be found for the quantities considered in this study, the runs of all three lengths were always pooled to increase the statistical power. Participants walked individually starting from seven different angles (+90°, +60°, +30°, 0°, −30°, −60°, −90°). The front edges’ midpoints of the starting areas were located within a 4 m radius to the center of the bottleneck crossing (Figure 1(left)). Furthermore, the width of the bottleneck was varied (0.4 m, 0.5 m, 0.6 m, 0.7 m, 0.8 m and 1.0 m), as well as the motivation (normal, hurried). For the ‘normal’ motivation, participants were instructed to ‘walk purposefully, but without haste’, and for the ‘hurried’ condition to ‘walk briskly, but not to run’. A table with all variables and increments has been published together with the raw data in the Pedestrian Dynamics Data Archive [31].

Participants were instructed to keep up their pace after crossing the bottleneck until they crossed a line four meters behind the bottleneck. This way, 36 experimental setups were tested (width × length × motivation). Each participant started from each starting angle once per setup, yielding 3276 runs (setups × participants × angles). Participants started walking in their own pattern and at their own pace, after the previous person had crossed the bottleneck, and always started their first step with the right foot. Instructions were repeated at least once before each setup.

**Figure 2:**
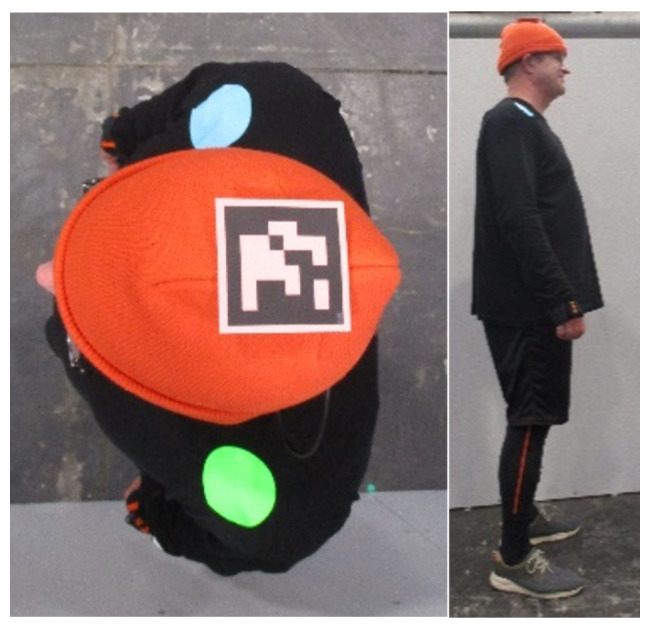
(**left**) Top view and (**right**) side view of participant equipped with orange hat, individual code, marked shoulders and motion capturing suit.

All participants were equipped with a combination of an orange hat and an individual Aruco code [33,34] for trajectory extraction from the video recordings, as well as a 3D motion capturing (mocap) suit [35,36] to track the full body motion (Figure 2). Furthermore, participants had colored markers on their shoulders, which are not part of the analysis presented in this paper. Data referring to the shoulders were obtained from the mocap suits. Two cameras (50 fps) with overlapping camera views were mounted under the ceiling (approximately 5.3 m above the floor), intended for the extraction of trajectories and documentation purposes.

## 3. Variables and Methods

### 3.1. Trajectories

The trajectory extraction was performed with the pedestrian tracking software PeTrack [2,32] for two overlapping camera views. The trajectories were checked and manually corrected if necessary. The camera views were combined into one single dataset by linear interpolation from the trajectory of one camera view to the trajectory of the other camera view in the overlap region. Head trajectories from camera data and mocap data were fused using a hybrid tracking algorithm [37]. After combination, fusion and visual inspection, 2840 trajectories (with 3D information at 60 Hz resolution) served for analysis.

In general, the walking path could be divided into three phases: (1) the acceleration phase (4 m to 3.5 m before crossing), (2) the straight walking phase (3.5 m to 1.5 m before crossing) and (3) the crossing phase (1.5 m before to 1.5 m after crossing). However, the values differed depending on whether the radius or the distance along the walking path was used as the basis of measurement.

### 3.2. Distance to Crossing

The ‘distance to crossing’ *d* is given as the distance along the COM trajectory. Positive values correspond to the distance to the crossing along the individual trajectory of a person, and negative values to the distance passed along the individual trajectory after crossing. The term ‘crossing’ refers to the point of the COM trajectory closest to y = 0 and is given as the position on the x-axis.

### 3.3. Calculation of Speed

The ‘mean speed’ was calculated for each person and run individually along the center of mass (COM) trajectory starting at a 3 m radius from the origin of the coordinate system to exclude the acceleration phase.

The ‘speed along path’ was calculated for each time step *t* with a centered running mean from the ‘distance to crossing’. To reduce the influence of steps, the window size was chosen to be 0.5 s, which referred to the approximate duration of one step. For the beginning and end of a trajectory, the time around *t* was decreased symmetrically, always including the first or last point. The maximum speed $v_{max}$ was calculated in the interval from the beginning of the straight walking phase until crossing (3.5 m to 0 m), and the minimum speed $v_{min}$ in the interval from the time of $v_{max}$ until the end of the walking path (Figure A2).

The ‘mean speed profiles’ were obtained by first calculating the mean speed for each speed profile in bins of 0.1 m between 4 m and −1 m individually and then calculating the mean for each bin.

### 3.4. Deceleration Point and Categorization of Deceleration

The deceleration point was determined from the smoothed acceleration curve of the COM trajectory. Contiguous data points with negative acceleration in the distance range of 3.5 to 0 m before the bottleneck were grouped and evaluated according to the duration and magnitude of the deceleration. A duration of one second was chosen as the temporal threshold and a deceleration of −0.0075 m/s^2^ (determined from 3σ interval during straight walking phase) as the amplitude threshold. When a group comprised a period of more than one second, the first group to which this applied was evaluated; otherwise, the first consecutive group of negative values was evaluated. The curves are distinguished as follows:

- ‘long & strong’: deceleration lasts **longer** than 1 s, with at least one data point of the group **larger** than the amplitude threshold;
- ‘short & strong’: deceleration lasts **less** than 1 s, with at least one data point of the group **larger** than the amplitude threshold;
- ‘long & weak’: deceleration lasts **longer** than 1 s, with **no** data point of the group larger than the amplitude threshold;
- ‘none’: deceleration lasts **less** than 1 s, and **no** data point of the group is larger than the amplitude threshold;
- ‘none at all’: no data point exists with negative acceleration before crossing the bottleneck.

**Figure 3:**
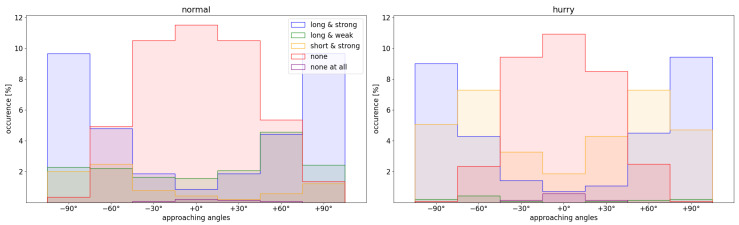
Histogram of frequency of occurrence of deceleration curves rated as ‘long & strong’, ‘short & strong’, ‘long & weak’, ‘none’ or ‘none at all’ depending on the starting angle. (**left**) For normal walking and (**right**) for hurried walking.

If the deceleration was classified as ‘long & strong’, ‘short & strong’ or ‘long & weak’, the first point of the group was selected as the deceleration point. If the deceleration was classified as ‘none’, the point was chosen as the deceleration point where a group of data points became negative for the first time and from this the first data point was selected. The frequency of occurrence of the different cases is shown in Figure 3. An example visualization of runs of the different categories is shown in the Appendix A (Figure A3).

### 3.5. Shoulder Rotation

**Figure 4:**
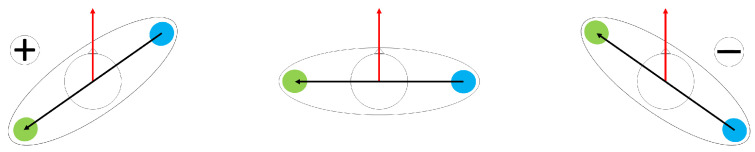
Schematic visualizing definition of shoulder rotation. The movement direction is indicated by the red arrow and the shoulderline by the black arrow. Blue and green circles symbolize colored shoulder markers of participants (c.f. Figure 2). Subfigures show (**left**) left turn, (**middle**) no rotation and (**right**) right turn.

The shoulder line was defined as the vector pointing from the right to the left shoulder (anat. acromion). The movement direction was defined as the vector between the positions within an interval of 0.5 s (corresponding to approximately one step) around the current COM position. The time interval was decreasing one-sidedly at the edges. Shoulder rotation γ(t) was defined as the angle between the ‘shoulderline’ and ‘movement direction’, rotated by 90°. This leads to γ = 0° if the shoulderline and movement direction are perpendicular to one another, positive values of γ in case of a left turn (turning towards the left shoulder/in the mathematically positive direction) and negative values of γ in case of a right turn (Figure 4).

The ‘mean rotation profiles’ were obtained by first calculating the absolute mean rotation for each rotation profile in bins of 0.1 m between 4 m and −1 m individually and then calculating the mean for each bin.

### 3.6. Determination of Onset and Maximum of Shoulder Rotation

The onset and maximum amplitude of shoulder rotation were derived from the shoulder rotation profiles γ(t). The ´onset’ is defined as the point in time where the rotation exceeds three times the standard deviation of straight walking (between 3.5 m and 1.5 m before crossing). The maximum shoulder rotation’ amplitude is defined as the maximum value of γ after the onset of rotation.

Mean values were calculated from the individually determined distances at which the onset of shoulder rotation or the maximum shoulder rotation occurred. Significance testing was performed by applying Tukey’s post hoc analysis at a 0.95 significance level.

Based on the curve progression of the data from [18], the data for the maximum shoulder rotation were fitted against an exponential function of the form $f\left(x\right)=ae^{-bx+c}+d$. The respective parameters with error bars are given in Figure A4. The ‘critical point’ $R_{crit}$ was calculated from the fit function f(x) for 180 evenly spaced values of *R* between 0.8 and 2.6, by applying a Tukey post-hoc analysis at a 0.95 level (analogous to [23]) for intervals of *R* of 0.05.

### 3.7. Shoulder Rotation and Corresponding Foot on the Floor

Step detection was performed based on the position data of the heels of the feet. The derivative (velocity) of the heels in the x and y directions was calculated and the sum of both was compared to a manually set threshold. Every time the slope exceeded the threshold, ground contact was identified and thus the beginning of a step. The corresponding foot on the floor was determined for the onset as well as the occurrence of the maximum rotation. It was defined as the foot on the floor that was closest in time before the occurrence of the onset or maximum. If the feet on the floor did not alternate in time, no foot was assigned as it could not be ruled out that floor contact was missed by the step detection algorithm.

## 4. Results and Discussion

In order to understand the space utilization of pedestrians when individually passing a bottleneck, different parameters were studied depending on the varied parameters of approaching angle, bottleneck width and walking speed. The parameters considered can be divided into two main themes: What space do individuals need when crossing a bottleneck, or where do they walk? How do individuals use the space available to them, or how do they move within it? The first question will be investigated in Section 4.2 and the second in Section 4.1, Section 4.3 and Section 4.4.

### 4.1. Speed and Start of Deceleration

**Figure 5:**
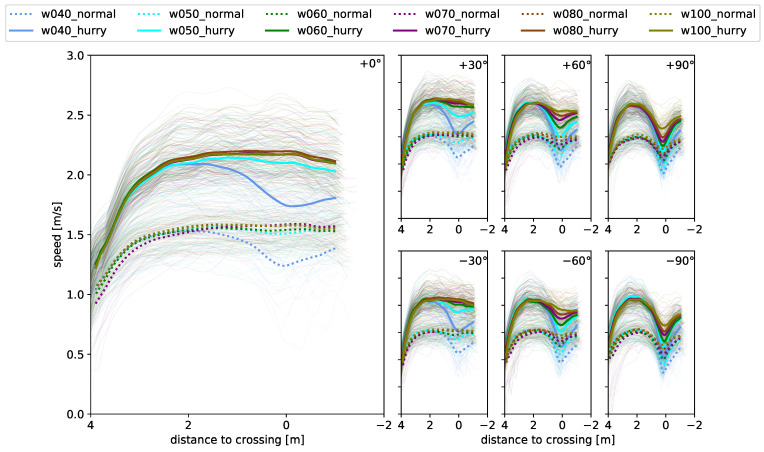
Speed profiles as a function of the distance to crossing the bottleneck. Subfigures show profiles for different starting angles: (left) 0°, (**upper**, left to right) +30°, +60°, +90° and (**lower**, left to right) −30°, −60°, −90°. Thick lines denote mean profiles calculated from individual profiles plotted as thin lines. Dotted lines denote runs under normal walking conditions; solid lines denote runs under hurried walking conditions. Colors denote bottleneck widths.

Speed can be considered a measure of how people move in the space available to them. Figure 5 shows the function of speed along the walking path for all starting angles (one per subplot) as well as bottleneck widths (color) and motivations (linestyle). The expected general shape of the functions shows an increase in speed during the acceleration phase, approximately 4 to 3 m before the crossing at x = 0, as well as a levelling off when reaching the preferred walking speed after 1 to 2 m of walking. In general, it can be noted that the two speed conditions, ‘normal’ and ‘hurried’, are clearly separated. The mean maximum speed attained was 2.1 m/s ± 0.7 m/s (median ± 2σ) for hurried and 1.5 m/s ± 0.6 m/s for normal walking and was roughly identical for all approaching angles. The distance to crossing at the point of $v_{max}$ was roughly constant at 2.0 m before crossing for large approaching angles and shifted towards 0.8 m before crossing for smaller angles and larger bottleneck widths (Figure A2(left)).

**Figure A1:**
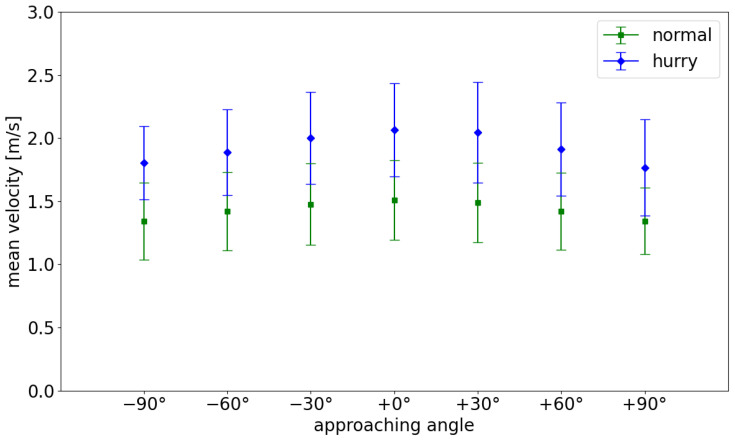
Mean velocity values for approaching angles as shown in Figure 1(left). Green squares denote data of normal walking speed condition and blue diamonds of hurried walking condition. Whiskers denote 2σ interval.

**Figure A2:**
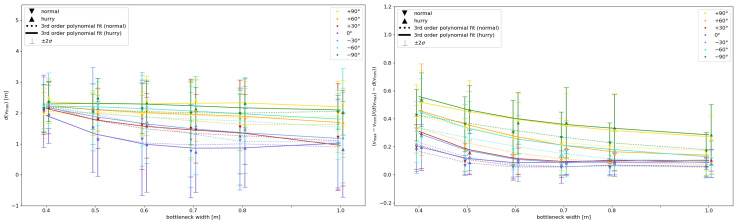
Upward pointing triangles denote data of hurried speed condition, downward pointing triangles denote data of normal walking speed condition. Colors denote approaching angle, whiskers show the 2σ interval. Solid/dashed lines show third-order polynomial fit for hurried/normal walking, respectively. Panels show (**left**) distance to crossing at time of $v_{max}$ against bottleneck width and (**right**) decline in speed from $v_{max}$ to $v_{min}$ over time for increasing bottleneck widths.

**Figure A3:**
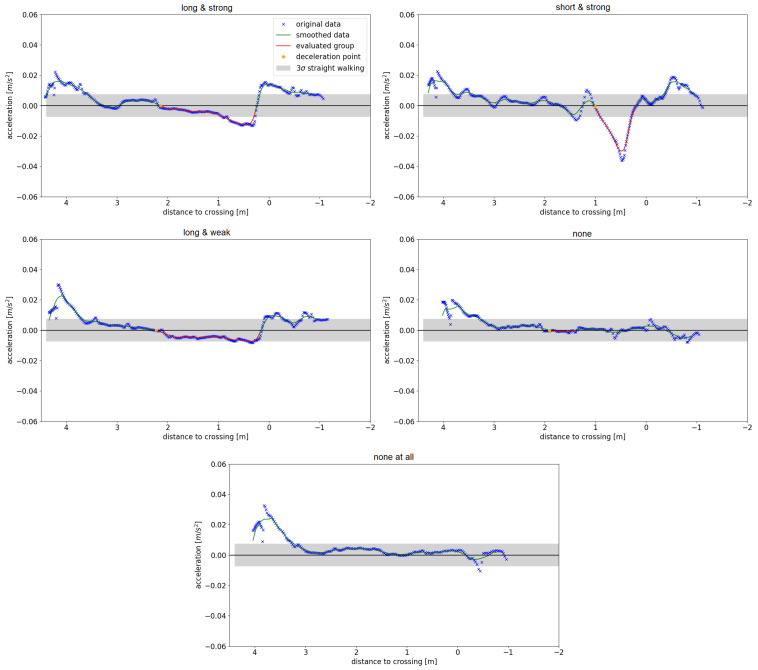
Exemplary deceleration curves from categories ‘long & strong’ (**upper**, **left**), ‘short & strong’ (**upper**, **right**), ‘long & weak’ (**middle**, **left**), ‘none’ (**middle**, **right**) and ‘none at all’ (**bottom**) as a function of distance to crossing. Blue crosses denote original data, green line denotes smoothed curve of original data, red line denotes evaluated group and grey shading denotes threshold calculated from 3σ of acceleration during straight walking phase. Orange dot denotes the deceleration point.

**Figure A4:**
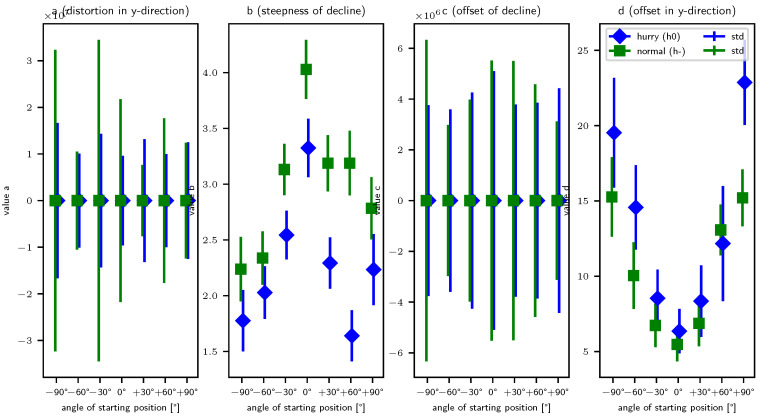
Parameters a, b, c and d of exponential fit function $f\left(x\right)=ae^{-bx+c}+d$, as mentioned in Section 3.

However, depending on the bottleneck width and approaching angle, the speed functions show different declines in speed after reaching the maximum speed. The decline is stronger for the hurried condition than for the normal walking condition and it is larger for a smaller bottleneck and larger approaching angle ( Figure A2(right)), yielding a mean maximum decline of 50% (hurried walking, w = 0.4 m, +90°). The observations are symmetrical for walking from the left and right (Figure A1). While slowing down can be observed for large approaching angles at all widths, it becomes less clear with straighter walking paths and larger bottleneck widths. The distribution of the deceleration intensity (Figure 3) further confirms these observations for the entirety of the runs. The non-deceleration runs spread around straight walking, while the heavy braking cases (‘long & strong’,‘short & strong’) cluster towards the large angles. Furthermore, the hurried condition has proportionally more cases of heavy braking (64%) than the normal condition (40%), but fewer cases of no braking (34% compared to 44%).

**Figure 6:**
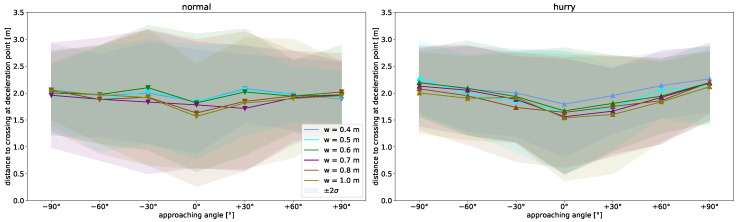
Start of deceleration as a function of starting angle (**left**) for normal walking and (**right**) for hurried walking. Colors denote bottleneck width. Markers indicate mean values. Shaded area denotes 2σ interval.

In contrast, a dependence on the angle for the distance when deceleration is started before the bottleneck is not observed for the normal condition (Figure 6(left)). The same applies to the dependence on the width of the bottleneck. For all starting angles and bottleneck widths, deceleration starts 2 m before crossing. For the hurried condition, a slight angle and width dependence can be seen (Figure 6(right)). However, this is only significant at a 0.95 level (Tukey’s post-hoc analysis) in the hurried condition between walking straight and the ±90° angles, as well as between the 0.4 m width and the widths of 0.7 m to 1.0 m.

### 4.2. Walking Path and Crossing Point

**Figure 7:**
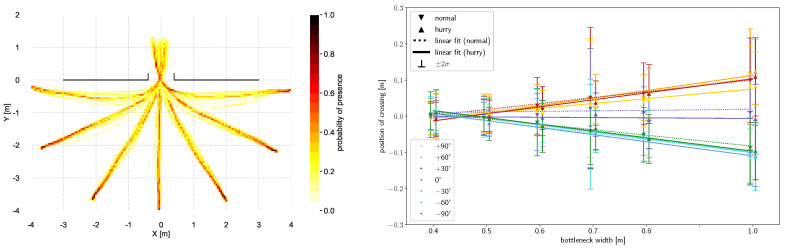
(**left**) Normalized heatmap showing probability of walking paths from COM trajectories for all seven starting angles, exemplarily for a bottleneck width of 0.5 m. (**right**) Crossing point as a function of bottleneck width calculated from COM trajectories. Triangles denote mean values for both speed conditions (up/hurried, down/normal) and are shifted apart for better visibility. Whiskers denote 2σ interval; colors indicate starting angles. The linear fit is shown in dotted lines for normal and in solid lines for hurried walking.

Another parameter to describe how individuals use a bottleneck is the walking path, as well as the point at which the bottleneck is crossed. Figure 7(left) shows the normalized probability of presence for all starting angles, for one bottleneck width example. When walking straight, a very straight walking path can be seen with little deviation to the sides. On the other hand, a wider spread of the walking path can be seen towards larger starting angles, which is also reflected in a greater variability of the position as well as the length of the walkway. Figure 7(left) shows differences with respect to the location of the crossing of the bottleneck.

The mean crossing points for all bottleneck widths and speed conditions are itemized in more detail in Figure 7(right). It is shown that the wider the bottleneck is, the further away from the center the bottleneck is crossed. The bottleneck is crossed further to the side from which the person originates. The offset is symmetrical around the straight walking position. Apart from walking straight, the angles do not differ; the same applies for different speeds. In the mean, the displacement towards the sides can be described by the following linear approximation:

C(w)=±0.176w∓0.078,

with *w* being the bottleneck width and C(w) the displacement on the x-axis. This means that the crossing point *C* shifts 1.76 cm towards the side of origin per 10 cm of increasing bottleneck width. With a wider bottleneck, more space is available that can be used by a person. Possible reasons for the displacement towards the sides could be the tendency to shorten the walking distance or a strategy to counteract drifting ‘outwards’ due to centrifugal force. In contrast, in [17], no displacement towards the bottleneck edges could be observed, which might be the case as the second bottleneck wall, which was not present in the cited study, served as visual guidance. At a bottleneck width of 0.4 m and 0.5 m, the bottleneck is potentially narrower than the shoulder width of the participant. Hence, the crossing point is largely determined by the direction in which a person turns to pass the obstacle. The possible preference of a direction of rotation (cf. Section 4.3) depending on the starting angle may counteract the observation described above in such cases.

### 4.3. When and How Are Shoulders Rotated?

In the following, it will be considered how the use of space in a bottleneck can be described by the rotation of the upper body. The shoulder rotation represents a significant measure of the space requirement of a person due to the spatial expansion of a person’s body in the horizontal plane. Therefore, it is also an important measure in pedestrian dynamics. In addition, the rotation of the upper body makes it possible to pass passages narrower than the shoulder width. The rotation behavior will be described in detail by (1) considering the time and place at which the rotation starts, (2) considering when the maximum amplitude is reached and (3) considering how the maximum amplitude is related to parameters such as the starting angle, velocity and bottleneck width. Rotations of other body segments, such as hip or head rotation, are also important for the description of the gait cycle and hence space utilization. However, as models in pedestrian dynamics are far from describing these parameters, they were not part of this analysis.

**Figure 8:**
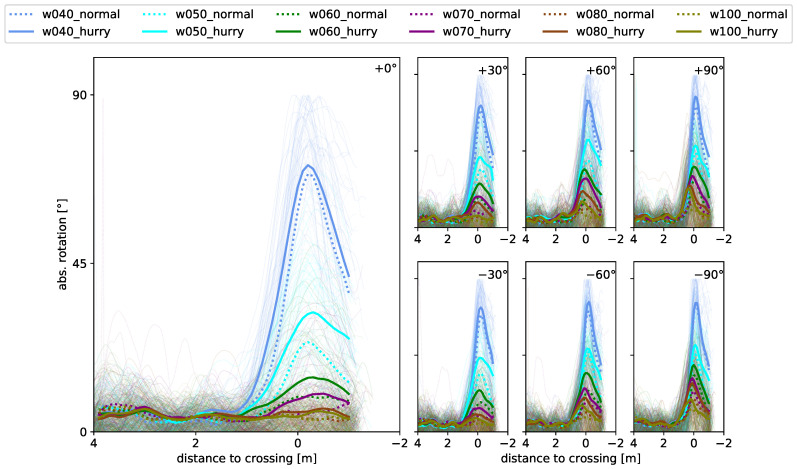
Absolute rotation profiles as a function of the distance to crossing the bottleneck. Subfigures show profiles for different starting angles: (left) 0°, (**upper**, left to right) +30°, +60°, +90° and (**lower**, left to right) −30°, −60°, −90°. Thick lines denote mean profiles calculated from individual profiles plotted as thin lines. Dotted lines denote runs under normal walking conditions and solid lines runs under hurried walking conditions. Colors denote bottleneck widths.

Figure 8 shows the function of absolute rotation along the walking path for all starting angles (one per subplot) as well as bottleneck widths (color) and motivations (linestyle). The curves show low rotation behavior of the shoulders during the straight walking phase, followed by a peak close to the crossing of the bottleneck. It furthermore shows more pronounced rotational behavior the smaller the bottleneck, as well as the greater the starting angle. The latter can be seen particularly clearly in the runs with a 0.8 m and 1.0 m bottleneck width, which show no clear peak for straight walking (0°) but an increasingly evident peak towards the larger approaching angles. The absolute maximum rotation amplitude, as well as its location and the location of the onset of rotation, will be described in more detail in the following.

**Figure 9:**
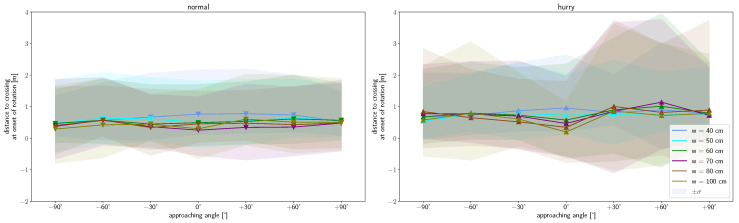
Distance to crossing at the onset of shoulder rotation as a function of approaching angle for normal (**left**) and hurried (**right**) walking. Markers show mean values and shaded area the 2σ interval. Colors denote respective bottleneck widths.

Figure 9 shows that rotation starts on average 0.49 m in front of the bottleneck at a normal speed for all starting angles. Meanwhile, in the hurried condition, rotation starts on average at 0.75 m and shows a slight right–left imbalance. The imbalance is reflected by a slight shift towards an earlier onset and higher variability when approaching from the right. Thereby, for the normal walking condition, only the 0.4 m and 0.5 m width runs are found to be significantly different from the runs of 0.7 m to 1.0 m. All widths in the hurried walking condition are found not to differ significantly in the mean. Only runs of the starting angle +60° and widths of 0.6 m and 0.7 m are significantly different from runs starting at 0° and widths of 0.7 m to 1.0 m (Tukey’s post-hoc analysis at 0.95 significance level). The same applies to the comparison of runs with a 0.4 m and 1.0 m bottleneck width and a starting angle of 0°. It is noticeable that the standard deviation is larger at starting angles of +30° to +90° in the hurried condition compared to the other starting angles and the normal walking condition. We speculate that this is due to a varying degree of right turn preference and associated locomotor adaptations in right-handers, which we would have expected to see much more frequently in this dataset. The distance of onset is in agreement with [25], who considered straight walking at bottleneck widths close to the shoulder width (corresponds to *w* = 0.4 m and 0.5 m). The mean time between the onset of rotation and crossing is similar (0.41 s and 0.46 s) for both speed conditions. Therefore, we assume that the backward-shifted onset is an adaption to the higher speed to account for the reaction time and the time needed for the top-down signal propagation of the body segments.

**Figure 10:**
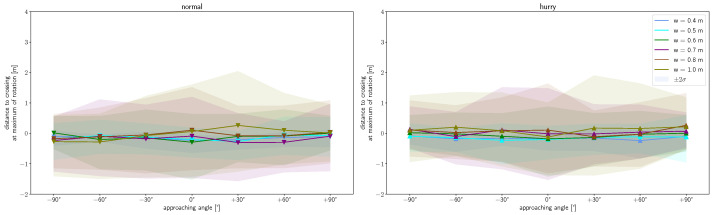
Distance to crossing at the time at which the maximum amplitude of shoulder rotation is exerted as a function of approaching angle for normal (**left**) and hurried (**right**) walking. Markers show mean values and shaded area the 2σ interval. Colors denote respective bottleneck widths.

Figure 10 shows that there is no angle or width dependence regarding the location of maximum rotation. However, a dependence of the speed can be noticed, since the occurrence of the maximum rotation at normal speed lies at an average of 0.09 m after the crossing, and, in the hurried condition, it is shifted slightly forward to 0.02 m after crossing. This is similar to the onset of rotation. The runs of the different widths do not differ significantly from one another, apart from runs at the 0.7 m compared to 1.0 m width at normal walking and runs at 0.5 m compared to all other widths with hurried walking.

**Figure 11:**
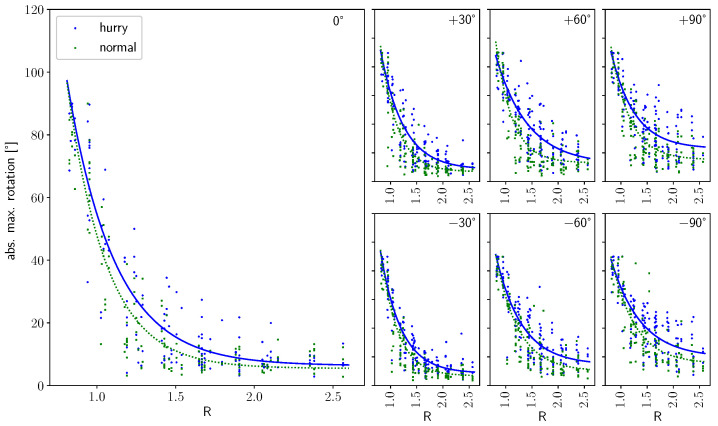
Maximum absolute shoulder rotation as a function of the ratio R=w/s. Subfigures show data points for different starting angles: (left) 0°, (**upper**, left to right) +30°, +60°, +90° and (**lower**, left to right) −30°, −60°, −90°. Solid lines show exponential fit for hurried and dashed lines for normal walking conditions. Markers show individual data points (green: normal, blue: hurried).

**Figure 12:**
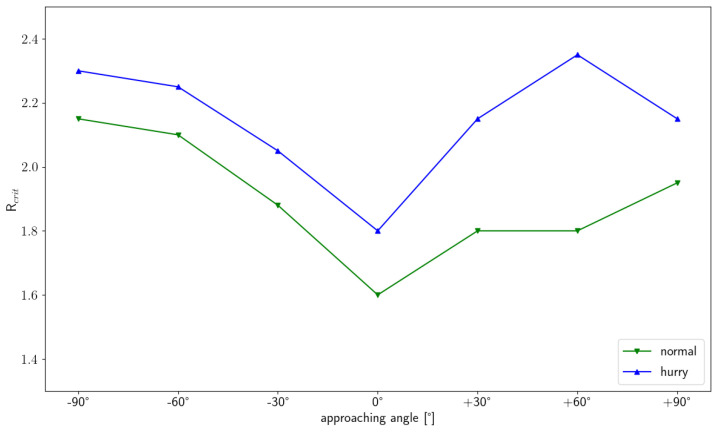
Critical point $R_{crit}$ as a function of approaching angle for normal (green) and hurried (blue) walking.

Figure 11 shows the amplitude of the maximum rotation for all starting angles (one per subplot) and motivations (color) against the bottleneck width scaled by the shoulder width of a person. For all parameters, it shows a decreasing course with a greater amplitude the smaller the ratio of the shoulder width to the bottleneck width or, in other words, the narrower the bottleneck. The progression continues until no change in amplitude is discernible, with an increasing ratio to very wide bottleneck widths. The difference that can be seen between the velocity conditions is that the amplitude at a normal velocity is always lower than that in the hurried condition. If we look at the differences between the starting angles, it can be seen that the amplitude does not drop to the same value at wider bottleneck widths as it does when walking straight ahead. Thus, at starting angles of 90°, a maximum rotation of 20° (normal) or 35° (hurried) can be observed even at large widths compared with an approach of 0° for straight walking. The fitted functions show that a change in the critical point would be expected between the two speed conditions, as well as between the starting angles. When determining the critical point $R_{crit}$ from the fitted curves (cf. Section 3), we find that the critical point depends on the starting angle as well as the speed condition (Figure 12). $R_{crit}$ is smallest for straight walking (1.6 for normal and 1.8 for hurried walking) and increases in a v-shaped manner towards the larger approaching angles. This means that the point from which the maximum shoulder rotation of pedestrians does not change for wider bottleneck widths shifts to wider bottleneck widths the more they have to move around the corner or the faster they walk.

The values are larger in general than the proposed critical value of [18] and more within the range of the critical values found for elderly pedestrians or if the bottleneck consisted of human beings by [22,23]. However, we know that the calculation based on a fit is not identical to the literature, as the bottleneck width was not set to fixed ratios of *w*/*s* settings per person, but to fixed bottleneck widths with a spacing of 10 cm, irrespective of the participant, due to the study design. If the critical point had been calculated analogously to the literature, without fitting, the error would have been out of range so that no meaningful comparison would have been possible. However, another interesting feature is the left–right asymmetry of $R_{crit}$ with respect to the approaching angle. Different interpretations are possible. Firstly, normal walking pedestrians, who are all right-handed, could be more used to turning rightwards and therefore exert rotations of smaller amplitude at smaller spaces. The general feature of smaller rotation amplitudes being reported the more trained a person is to the situation and speed condition has, for example, been shown in a comparison of professional vs. non-professional football and rugby athletes [19]. Another contribution to the asymmetry could be that the walking paths slightly differ in shape and therefore length, as can be gained as an impression from Figure 7(left) for the 90° angles, whereby a rotation adjustment is also to be expected. Furthermore, it is possible that the asymmetry is caused by adaptations of the locomotor system to the placement of the feet on the floor when starting the rotation or mitigating the course of the rotation. This could be produced as a systematic signal, as participants always started their first step with the right foot. Thus, participants would arrive at the bottleneck with a different ‘foot on the floor’ to the ‘intended movement direction’ setting when approaching from the right compared to the left, when not making locomotor adjustments.

### 4.4. Connection between Shoulder Rotation and Feet

**Figure 13:**
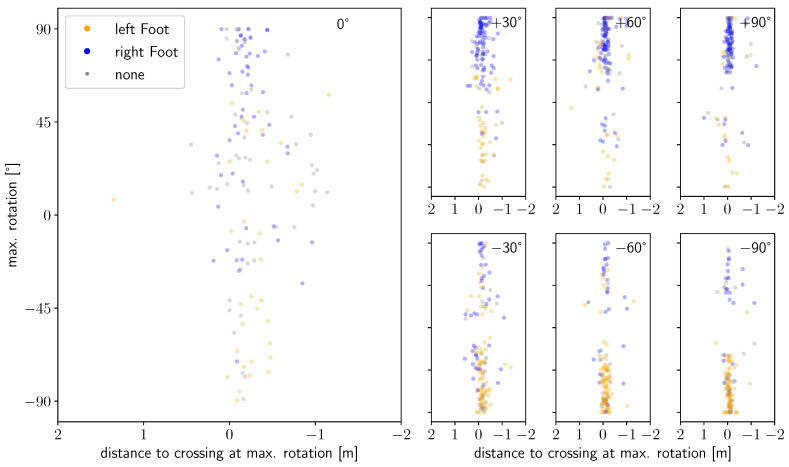
Scatterplot of maximum shoulder rotation as a function of the distance to crossing at the time of maximum rotation for runs with R≤1.3. Subfigures show profiles for different starting angles: (left) 0°, (**upper**, left to right) +30°, +60°, +90° and (**lower**, left to right) −30°, −60°, −90°. The foot on the floor at the time of maximum rotation is denoted by the color (orange: left, blue: right, grey: no foot).

**Figure A5:**
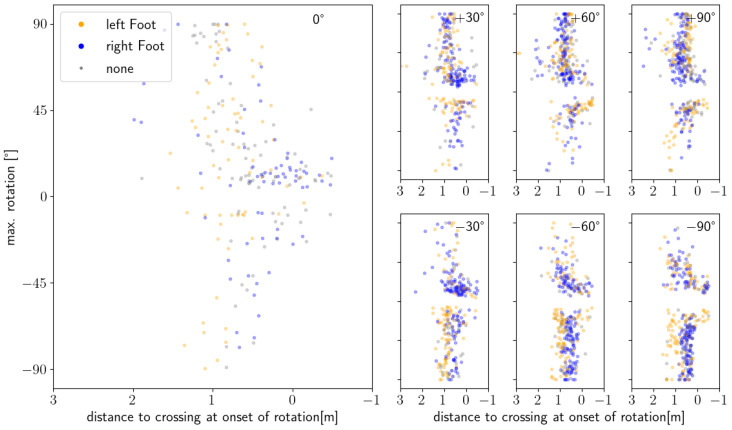
Scatterplot of maximum shoulder rotation as a function of the distance to crossing at the time of the onset of shoulder rotation for all runs. Subfigures show profiles for different starting angles: (left) 0°, (**upper**, left to right) +30°, +60°, +90° and (**lower**, left to right) −30°, −60°, −90°. The foot on the floor at the time of maximum rotation is indicated by the color (orange: left, blue: right, grey: no foot).

**Figure A6:**
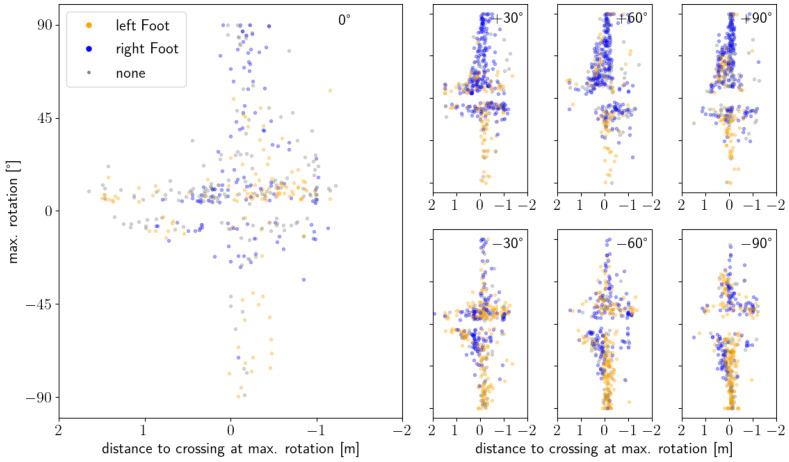
Scatterplot of maximum shoulder rotation as a function of the distance to crossing at the time of maximum rotation for all runs. Subfigures show profiles for different starting angles: (left) 0°, (**upper**, left to right) +30°, +60°, +90° and (**lower**, left to right) −30°, −60°, −90°. The foot on the floor at the time of maximum rotation is indicated by the color (orange: left, blue: right, grey: no foot).

Lastly, we consider the connection between shoulder rotation and the second variable that influences space utilization in the movement plane, which is the feet. In particular, we wish to investigate the hypothesis that the foot that is on the ground at the time of executing a movement influences or even determines the amplitude and direction of the shoulder rotation. In other words, we expect a preferred direction of rotation depending on the starting angle, but this direction would be reversed should the person arrive at the bottleneck with the ‘wrong’ foot for him/her. Figure 13 shows the distribution of the maximum rotation amplitude against the position at which the maximum rotation is executed. The markers are colored according to the respective foot on the floor. The figure comprises data points for experimental conditions where rotation of the shoulders would be necessary, as expected by the literature value of R ≤ 1.3. It shows an equal distribution of right and left turns for straight walking, as well as more left turning compared to right turning, when starting from the right (+90°) and vice versa for starting from the left side (−90°) of the bottleneck. Furthermore, the strong accumulation of ‘right’ feet on the floor for left turns (80% at +90°, 76% at +60° and 68% at +30° of the datapoints with assigned feet) and ‘left’ feet on the floor for right turns (80% at −90°, 75% at −60° and 66% at −30°) is apparent, which confirms our hypothesis. The connection is probably due to the anatomy or gait rhythm, i.e., the dependence of shoulder rotation on the gait phase. However, this observation is not as clearly visible in the diagram of maximum rotation, where rotation is not necessarily expected (cf. Figure A6). In cases where rotation is not necessary, lower amplitudes of rotation occur (Figure 11) in general and maximum rotations are executed at a broader range before and after crossing. Therefore, rotation profiles where rotation is not necessarily expected are more likely to show the opposite sign/foot combination compared to rotation profiles where rotation would be expected for the same starting angle. Moreover, no correlation is visible for the amplitude or direction of rotation with the position of the onset of rotation or the foot on the floor at the onset of rotation itself (cf. Figure A5). This applies both to runs where rotation would be expected and to runs where this is not the case. Only a small signal can be identified indicating that if the onset occurs before approximately 0.6 m before crossing, the opposite foot is on the ground compared to the case in which the onset occurs later than 0.6 m before crossing. This can be explained by the fact that people, depending on their step length, are able to perform either three or four steps by the time they reach the bottleneck.

**Figure 14:**
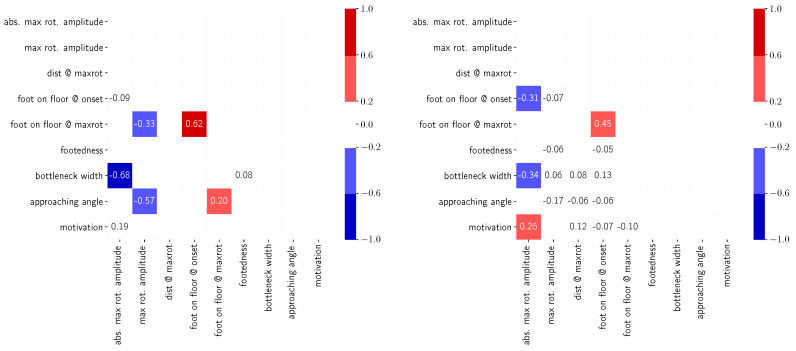
Heatmap of correlation matrix between parameters connected to the rotation process for data where (**left**) *R* ≤ 1.3 and (**right**) *R*> 1.3. Values that are not significant on a 95% level are not displayed. Color coding highlights the magnitude of correlation.

These observations are also verified by the correlation matrix shown in Figure 14. The correlation matrix is divided into two parts for the purpose of a rotation-dependent discussion. On the left, parameters are compared for experimental runs where shoulder rotation would be expected from the literature [18] (R≤ 1.3) and on the right where shoulder rotation is not necessary (*R*> 1.3).

For both cases, a strong, significant correlation can be seen between the foot on the ground at the onset of rotation and the foot on the ground when reaching the maximum amplitude of shoulder rotation. This is due to the fact that, in most cases, one step is taken between the two points in time, as the distance between onset and exerting the maximum amplitude remains roughly constant (Figure 9 and Figure 10). For experimental runs in which, according to the literature, no shoulder rotation is to be expected, weak correlations exist for the absolute maximum shoulder rotation amplitude with (1) the bottleneck width, (2) the motivation (speed) and (3) the foot on the floor at the onset of rotation, which supports the observations from the previous sections. For experimental runs in which shoulder rotation is to be expected, the correlations support the previously reported observations that (1) the bottleneck width influences the amplitude of shoulder rotation, while (2) the approaching angle influences the direction of rotation. Furthermore, the correlations confirm that the maximum rotational amplitude is influenced by the foot on the floor at the time of maximum rotation. The correlation value appears to be weaker than the visual perception from Figure 13, as probably non-determined feet reduce the value mathematically. We consider correlations of >0.3 as not relevant and therefore do not further discuss the comparison between the foot on the floor at the time of maximum rotational amplitude with the approaching angle. Furthermore, no correlation between the width of the bottleneck and the start or timing of the maximum rotation can be determined, which supports the observations from the previous paragraph. Moreover, no correlation between footedness and feet on the ground at either onset or maximum rotation can be seen. However, this could also generally be due to the short walking distance of 4 m, in which no great variation in the number of steps is possible and thus right and left footers (50:50 in the study) statistically balance each other out.

## 5. Conclusions and Outlook

In this study, we investigated whether time- and location-dependent generalizable movement patterns exist that describe the space requirements and use of space of an individual person when passing a bottleneck. For this purpose, the walking path with a crossing point, the speed, the deceleration behavior and the shoulder rotation and foot position were investigated as a function of the starting angle, bottleneck width and walking speed. The results can be used to steer or validate the speed of agents in models within free walking conditions.

We find that the walking distance is mainly modulated by the width of the bottleneck and minimally by the starting angle or walking speed. Thus, the crossing point shifts in the mean by 1.75 cm in the direction from which the person is approaching for each 10 cm increase in bottleneck width. The speed profiles and the mean velocity, on the other hand, strongly depend on the approaching angle. The differences between the maximum and minimum speed values arise mainly from the different distributions of deceleration patterns (more cases of heavy braking for the hurried condition) and less from the starting point of deceleration, which can be considered invariant for all starting angles and both velocity conditions. The maximum amplitude of the shoulder rotation strongly depends on the starting angle and the ratio of the bottleneck width to the shoulder width of the participants. We found that the ‘critical point’ $R_{crit}$ = 1.3, used to ensure shoulder rotation in a number of studies, is not a constant value but rather increases for larger approaching angles as well as with the walking speed. Therefore, one needs to keep in mind that, when using this widely adopted value to distinguish scenarios where rotation is necessary or not necessary, runs that, strictly speaking, fall into the category of ‘rotation is expected’ are also included in the category ‘rotation is not needed’. The distance to the crossing at the start of the rotation, as well as at the time of the maximum rotation, is constant per speed condition and independent of the bottleneck width and starting angle. The distance to the crossing at the beginning of the rotation, as well as at the time of the maximum rotation, is constant for each speed condition and independent of the width of the bottleneck and the starting angle. Furthermore, it has been shown that for experimental runs in which a shoulder rotation is to be expected, the direction of rotation is significantly influenced by the foot on the ground at the time of the maximum rotation (usually co-occurring with the foot at the beginning of the rotation), which is likely connected to the gait rhythm. Thus, the rotation is mostly performed as a left turn when the right foot is on the ground and as a right turn when the left foot is on the ground.

The strong correlation between the approaching angle, bottleneck width and amplitude/direction of rotation shows that more effort should be put into understanding and utilizing the individual motion of people for pedestrian dynamics, to point out easy-to-model relationships and to be able to implement them in models of pedestrian dynamics in the future. Based on the findings of this study, it would be of interest to investigate to what extent movement and space utilization change when the presence of other persons influences the available space and the individual possibilities of action. It is recommended to investigate the effect of how the presence of other persons, as well as the interpersonal interactions between them, changes the temporal and spatial use of space and whether generalizable correlations can be shown with the suitable grouping of behavior patterns or strategies.

## Figures and Tables

**Table 1:** Participant statistics for age, body height, gender, shoulder width and footedness.

		Median	Standard Deviation
age	[years]	31.0	8.6
body height	[m]	177.0	9.4
gender	[no. of female/male]	7/6	
shoulder width	[cm]	43.0	3.3
footedness	[no. of right/left]	7/6	

## Data Availability

Data are published and openly available at Forschungszentrum Jülich, Institute for Advanced Simulation: Data Archive of Experiments on Pedestrian Dynamics https://doi.org/10.34735/ped.2020.3 [31] (accessed on 9 January 2024).

## Appendix A

This appendix contains additional material and diagrams that include the values mentioned in the main text in the context of other parameters or state numbers to the fitted data.

### Appendix A.1. Additional Material for Speed Considerations

Figure A1 shows a symmetric dependence of the mean speed with respect to the approaching angle as mentioned in Section 4.1. The mean is calculated over all bottleneck widths and lengths. The error bars of the speed conditions overlap, since participants were able to choose their own speed to match the announcement. Possible reasons for the mean speed differences between the participants could include, but are not limited to, body size (stride length), physical fitness, cultural background and inner posture. The data points of fast participants are always situated in the upper and data points of slow participants always in the lower part of the respective speed condition. Since the patterns of coordination change depending on the self-selected speed [11], this is assessed to be a desirable behavior.

Figure A2(left) shows the data for the distance to crossing at the time of maximum speed, as mentioned in Section 4.1. The data are fitted against a third-order polynomial function of the general shape $f\left(x\right)=ax^{3}+bx^{2}+cx+d$. Fitted values for a, b, c and d are shown in Table A1. Figure A2(right) shows data for the decline in speed from the $v_{max}$ to $v_{min}$ over time for increasing bottleneck widths, as mentioned in Section 4.1. The data are fitted against a third-order polynomial function of the general shape $f\left(x\right)=ax^{3}+bx^{2}+cx+d$. Fitted values for a, b, c and d are shown in Table A1.

### Appendix A.2. Additional Material for Shoulder Rotation

Figure A4 shows the parameters of the exponential fit function of the form $f\left(x\right)=ae^{-bx+c}+d$, as mentioned in Section 4.1.

**Table A1:** Parameters a, b, c and d of binomial fit function $f\left(x\right)=ax^{3}+bx^{2}+cx+d$, as mentioned in Section 4.1.

	a	b	c	d
$d(v_{max})$				
h + 90°	0.00668696	−0.07988463	0.24201051	1.80331215
h + 60°	0.00642178	−0.06637546	0.09262251	2.02087564
h + 30°	−0.00934236	0.14740765	−0.83550713	2.92320077
h + 0°	−0.01960144	0.28470059	−1.33211802	2.99259806
h − 30°	0.01024136	−0.08627944	−0.0882005	2.39037012
h − 60°	−0.00490639	0.06462839	−0.34151718	2.44439803
h − 90°	−6.09631951 ×$10^{-5}$	1.48160548 × $10^{-2}$	−1.40612429 ×$10^{-1}$	2.33404726
h0 + 90°	−0.00534329	0.06027801	−0.2044605	2.5090688
h0 + 60°	−0.00406905	0.05005988	−0.26035428	2.45298494
h0 + 30°	−0.01116662	0.14499333	−0.72207756	2.73009221
h0 + 0°	−0.01090344	0.19848113	−1.11460665	2.84074908
h0 − 30°	−0.00279493	0.05477439	−0.44655222	2.57362941
h0 − 60°	1.75176559 × $10^{-3}$	−2.62980560 ×$10^{-2}$	3.95093048 × $10^{-2}$	2.21138498
h0 − 90°	0.00351195	−0.04646964	0.13940715	2.19156437
$\left(v_{max}-v_{min}\right)/\left(d\left(v_{max}\right)-d\left(v_{min}\right)\right)$				
h + 90°	−0.00069317	0.01591055	−0.1295903	0.53734671
h + 60°	−0.0011692	0.02418756	−0.17024772	0.48156084
h + 30°	−0.00353316	0.05126638	−0.23427057	0.4004375
h + 0°	−0.00312153	0.04378574	−0.19050351	0.31678929
h − 30°	−0.00299053	0.04510905	−0.21988483	0.40853455
h − 60°	6.34530468 × $10^{-4}$	−2.47684664 ×$10^{-4}$	−7.36817531 ×$10^{-2}$	4.08661745 × $10^{-1}$
h − 90°	4.30221584 × $10^{-4}$	−1.50664587 ×$10^{-3}$	−5.35025696 ×$10^{-2}$	4.79013964 × $10^{-1}$
h0 + 90°	−7.86952067 ×$10^{-5}$	5.80993692 × $10^{-3}$	−8.32584043 ×$10^{-2}$	5.97788653 × $10^{-1}$
h0 + 60°	1.65880374 × $10^{-4}$	8.04219200 × $10^{-3}$	−1.27093673 ×$10^{-1}$	5.78284074 × $10^{-1}$
h0 + 30°	−0.00316142	0.05043629	−0.25765869	0.52087808
h0 + 0°	−0.0035431	0.04853971	−0.20521354	0.36073896
h0 − 30°	−0.00276978	0.04506278	−0.2364581	0.48876656
h0 − 60°	−0.0020037	0.03290115	−0.20292201	0.62379791
h0 − 90°	−0.00139271	0.02355754	−0.1547143	0.69154799

### Appendix A.3. Additional Material for Deceleration Cases

Figure A3 provides exemplary curves of deceleration categorized as ‘long & strong’, ‘short & strong’, ‘long & weak’, ‘none’ or ‘none at all’, as well as the resulting calculated deceleration point (orange dot). In total, 889 curves were categorized as long & strong, 581 as short & strong, 251 as long & weak, 1097 as none and 19 as none at all.

Figure A5 shows the maximum shoulder rotation as a function of the distance to crossing at the time of the onset of shoulder rotation for all runs. In the main text, this figure is referenced to visualize the non-existing correlation between the foot on the floor and the rotation amplitude or sign. However, as mentioned, the data suggest that there is a connection between the distance to crossing at the onset of rotation and the respective foot on the floor (e.g., subplot −90°) as there is a clear separation of the foot on the floor before and after approximately 0.6 m before crossing.

For completeness, Figure A6 shows the maximum shoulder rotation as a function of the distance to crossing at the time of maximum rotation for all runs, compared to Figure 13 in the main text, which only shows runs for *R* ≤ 1.3.
